# Molecular Dissipative Structuring: The Fundamental Creative Force in Biology

**DOI:** 10.3390/e28020246

**Published:** 2026-02-20

**Authors:** Karo Michaelian

**Affiliations:** Department of Nuclear Physics and Application of Radiation, Instituto de Física, Universidad Nacional Autónoma de México, Circuito Interior de la Investigación Científica, Cuidad Universitaria, Mexico City CP 04510, Mexico; karo@fisica.unam.mx

**Keywords:** origin of life, abiogenesis, non-equilibrium thermodynamics, molecular dissipative structuring, thermodynamic dissipation theory, prebiotic chemistry, natural selection, thermodynamic selection, biosphere, 92C05, 92C15, 92C40, 92C45, 80Axx, 82Cxx, 82B35, 82C26

## Abstract

The spontaneous emergence of macroscopic dissipative structures in systems driven by generalized chemical potentials is well established in non-equilibrium thermodynamics. Examples include atmospheric/oceanic currents, hurricanes and tornadoes, Rayleigh–Bénard convection cells and reaction–diffusion patterns. Less well recognized, however, are microscopic dissipative structures that form when the driving potential excites internal molecular degrees of freedom (electronic states and nuclear coordinates), typically via high-energy photons or coupling with ATP. Examples include dynamic nanoscale lipid rafts, kinesin or dynein motors along microtubules, and spatiotemporal Ca^2+^ signaling waves propagating through the cytoplasm. The thermodynamic dissipation theory of the origin of life asserts that the core biomolecules of all three domains of life originated as self-organized molecular dissipative structures—chromophores or pigments—that proliferated on the Archean ocean surface to absorb and dissipate the intense “soft” UV-C (205–280 nm) and UV-B (280–315 nm) solar flux into heat. Thermodynamic coupling to ancillary antenna and surface-anchoring molecules subsequently increased photon dissipation and enabled more complex dissipative processes, including photosynthesis, to dissipate lower-energy but higher-intensity UV-A and visible light. Further thermodynamic coupling to abiotic geophysical cycles (e.g., the water cycle, winds, and ocean currents) ultimately led to today’s biosphere, efficiently dissipating the incident solar spectrum well into the infrared. This paper reviews historical considerations of UV light in life’s origin and our proposal of UV-C molecular dissipative structuring of three classes of fundamental biomolecules: nucleobases, fatty acids, and pigments. Increases in structural complexity and assembly into larger complexes are shown to be driven by the thermodynamic imperative of enhancing solar photon dissipation. We conclude that thermodynamic selection of dissipative structures, rather than Darwinian natural selection, is the fundamental creative force in biology at all levels of hierarchy.

## 1. Introduction

There are two kinds of structures in nature: equilibrium structures resulting from the maximization of entropy (e.g., minimization of the Gibbs potential at constant temperature and pressure), and non-equilibrium structures (or “processes”, since these are often dynamical) resulting from the optimization of entropy production. Examples of equilibrium structures include crystalline structures and the solar system. Examples of non-equilibrium structures include hurricanes, Bénard cells, reaction–diffusion patterns, the water cycle and atmospheric/oceanic currents. These latter structures were given the name “dissipative structures” by Ilya Prigogine [1,2,3], who, building on the work of Lars Onsager [4,5,6], developed the mathematical formalism for treating them in the latter half of the 20th century.

Although macroscopic dissipative structures, such as the examples given above, are well known, microscopic dissipative structures are less well known. These are formed through the excitation of internal molecular degrees of freedom (electronic states and nuclear coordinates). Examples include ATP-driven molecular motors (kinesin, myosin walking on tracks) [7,8], flagellar motors (run-and-tumble dissipation of proton gradient) [9,10], and protein production in the rybozyme driven by chemical potential [11].

We have identified a previously unrecognized class of microscopic dissipative structures fundamental to the origin of life: organic UV-C chromophores or pigments, dissipatively structured under the UV-C light flux at the Earth’s Archean surface from common precursors such as hydrogen cyanide (HCN), cyanogen (NCCN) and carbon dioxide (CO_2_) in water [12,13,14]. These structures effectively absorb and convert high-energy photons into many lower-energy photons, thereby performing the non-equilibrium thermodynamic imperative of entropy production. Today, these UV-C chromophores are known as the fundamental molecules of life because they are in the three domains of life (archaea, bacteria and eukaryote) and therefore at the very foundations of life.

**Figure 1:**
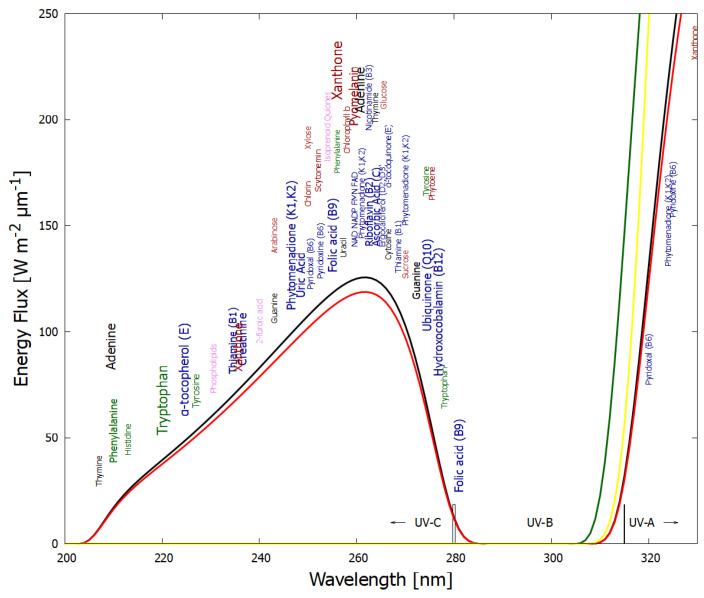
The spectrum of UV light available at the Earth’s surface before the origin of life at approximately 3.9 Ga and until at least 2.9 Ga (black and red curves, respectively). The spectrum in the UV-C may even have persisted throughout the entire Archean until 2.5 Ga [16]. Atmospheric CO_2_, H_2_O, SO_2_ and probably some H_2_S were responsible for the absorption of wavelengths shorter than ∼205 nm, and atmospheric aldehydes (e.g., formaldehyde and acetaldehyde, common photochemical products of CO_2_ and water) absorbed between about 285 and 305 nm [17,18], approximately corresponding to the UV-B region (280 and 315 nm). By around 2.2 Ga (green curve), UV-C light at Earth’s surface was completely extinguished by oxygen and ozone (which we consider as life-produced UV-C pigments) resulting from organisms performing oxygenic photosynthesis. The yellow curve corresponds to the present surface spectrum. Energy fluxes are for the Sun at the zenith. Over 50 fundamental molecules of life are plotted at their wavelengths of maximum absorption: nucleic acids (black), amino acids (green), fatty acids (violet), sugars (brown), vitamins, co-enzymes and cofactors (blue), and pigments (red). We have asserted that these fundamental molecules were dissipatively structured as UV-C pigments under this light. The font size is roughly proportional to the relative size of the respective molar extinction coefficient of the pigment. Reproduced with permission from Michaelian [19].

These fundamental chromophores absorb very strongly around the peak in the incident UV-C spectrum arriving at Earth’s surface during the Archean (Figure 1) and dissipate the absorbed energy very rapidly into heat through conical intersections [15] (Section 4), endowing them with a wide wavelength absorption bandwidth. They include nucleotides, amino acids, fatty acids, sugars, vitamins, coenzymes, cofactors, and pigments. Although today they have different functions, most still retain their extraordinary UV-C absorption and dissipation properties, probably an indication of the length of time that life was subjected to this light, from life’s origin to before the existence of an ozone layer (∼1400 million years).

This review describes dissipative structuring in life, from the primordial (Archean) formation of fundamental molecular structures under UV-C photon flux to today’s complex biosphere. First, a brief historical perspective on the discovery of the utility of UV light for abiogenesis is given. Next, an introduction to the classical irreversible thermodynamic (CIT) theory of Prigogine and collaborators [2] in the non-linear regime is provided. Then, the thermodynamics and dynamics of molecular dissipative structuring of chromophores occurring at the origin of life under the Archean surface UV-C light are described using three examples: nucleobases, fatty acids, and pigments. Evolutionary increases in the complexity of these structures are shown to be driven by the thermodynamic imperative of increasing photon dissipation. Finally, we compare evolution through thermodynamic selection of entropy production by dissipative structuring to evolution through natural selection proposed in the Darwinian and Gaia theories. We conclude that thermodynamic selection on dissipation, including both stochastic and deterministic elements, rather than Darwinian natural selection, is the true fundamental creative force in biology.

## 2. A Historical Perspective

Carl Sagan speculated in 1957 [20] that, as a result of the lack of an ozone layer, UV light at Earth’s surface during the Archean may have played a dual role in abiogenesis: synthesis and degradation of organic compounds. Around the same time, it was being established that UV-C light could cause structural damage to DNA, including strand breaks, deletions, and covalently bonded products among adjacent nucleobases (e.g., cyclobutane pyrimidine dimers, CPDs). In a later publication [17], Sagan suggested that incipient life must, therefore, have been subjected to a strong form of natural selection pressure under this light. He determined that unprotected organisms would receive lethal UV doses in under 0.3 s and proceeded to propose mechanisms of early Archean photoprotection.

The idea of incipient life acquiring UV photoprotection, whether by “design” through natural selection of photo-stability, or through external shielding, thus became a common theme in origin of life research. Examples of active areas of research include photoprotective pigments and proteins [21,22,23,24,25,26], quenching of molecular excited states through internal conversion [27,28,29], vesicle shielding through UV Mie scattering [30], UV-shielding mineral cavities or solutions [31,32], and deep sea hydrothermal vent origin-of-life scenarios [33] (Mulkidjanian et al. [34] compare surface geothermal fields with deep-sea hydrothermal vents as cradles for the origin of life).

Molecular synthesis under UV light has received similar attention. Experiments with UV light on different precursors have led to the synthesis of small but detectable amounts of nucleobases [35,36,37,38,39,40,41,42], amino acids [42,43], fatty acids [42] and some sugars [44,45]. Experiments probing the utility of UV light for the synthesis of complexes of fundamental molecules (e.g., nucleoside formation and phosphorylation) have also had success [46,47,48,49]. Furthermore, the utility of UV light in biasing particular chemical reaction networks has been investigated [50]. Some of these results have been supported with time-dependent quantum mechanical calculations mapping out the excited state potential energy surface en route to the molecules [27,51].

Beginning in 2009 [12,13], we published a series of papers proposing that the fundamental molecules of life were UV-C photon dissipative structures (pigments or chromophores), “designed” through non-equilibrium thermodynamic directives to dissipate the UV-C photons into heat. The free energy available in the photon was, of course, a useful component for overcoming high-energy conformational barriers in synthesis, but the important thermodynamic variable promoting structuring and accumulation to more than the nominal concentration was entropy production through photon dissipation. The photon-dissipative nature of the fundamental molecules was thus seen not as an auxiliary protective feature, but rather as the thermodynamic design goal, the only physical–chemical reason for their abiogenesis and build-up to large concentrations during the Archean. A corroborating fact is that strong broadband UV-C molecular absorption is not required for photoprotection or even synthesis, but it is for photodissipation.

The “thermodynamic dissipation theory for the origin and evolution of life” (TDTOL) asserts that life arose as spontaneous molecular dissipative structuring of organic chromophores (pigments) to dissipate the incident UV-C solar light flux available at Earth’s surface throughout the Archean. This led to a primordial “UV-C Pigment World” derived from common carbon-based precursors such as HCN, NCCN, and CO_2_ at the ocean surface under soft UV-C (205 to 285 nm) and soft UV-B (310–320 nm) light [52].

Since the first papers outlining the thermodynamic principles of the theory [12,13,19,53], we have addressed, from within this photon dissipative perspective, many important problems concerning the origin of life, including molecular synthesis through dissipative structuring [14,54,55,56,57], enzymeless RNA/DNA replication [58,59], homochirality [60], amino acid codon assignments [61], and fatty acid synthesis for vesicles [14,30,55]. The treatment of such diverse and fundamental problems from within within a single conceptual framework has not previously been achieved.

The theory further provides an explanation [62] for UV-absorbing organic molecules found on other planets [63,64], comets [65], meteorites [66,67] and in interstellar space [68,69], giving rise, for example, to the galactic 217.5 nm UV extinction bump [70].

## 3. Non-Linear Classical Irreversible Thermodynamic Theory

Classical irreversible thermodynamic (CIT) theory, developed by Théophile de Donder, Lars Onsager, Ilya Prigogine, Paul Glansdorff, Grégoire Nicolis, Agnessa Babloyantz, and others [1,2,3] from the “Brussels school”, has proven to be a very useful formalism for understanding living systems, including the origin of life [12,13,54,55,59,60,61,71,72,73], the cell [30,74], cell differentiation [75], cognition [76,77], ecosystems [78,79], the biosphere [3,74,80,81,82] and even the synthesis of organic molecules detected in space [62].

Within CIT theory, processes are driven by what are known as *generalized chemical potentials*. Examples include the electric potential which promotes the movement of charged material (a current), temperature potentials which promote a flow of energy (heat flow), concentration potentials which promote the flow of matter (diffusion), chemical and photochemical potentials which promote molecular transformations (chemical and photochemical reactions), etc. The potentials divided by the ambient temperature are known as *generalized thermodynamic forces* because the size of these quantities determines the strength of the corresponding *generalized thermodynamic flows* (e.g., heat flow, diffusion, or reaction rate). The entropy production of a system can be shown to be just the sum of the products of all the forces times their respective flows [2].

When thermodynamic forces are sufficiently large, the relation between force and flow is no longer necessarily linear. Internal forces can lead to new internal flows and so on, giving rise to a set of coupled and even catalytically coupled processes and, therefore, non-linear behavior. It is precisely this non-linearity between forces and flows that leads to numerous possible (stable and unstable) stationary solutions for the system [2,3] and thus the large diversity of dynamics seen in the interaction of material with its environment, especially for carbon-based materials when subjected to a strong photochemical potential, a scenario which we call “life”.

The elements of non-linear CIT theory describing the dynamics and evolution of a material system held far out of equilibrium through interactions of the system with its environment are the following [83];

1. The existence of at least one relatively constant applied external generalized thermodynamic potential defining the environment—the applied thermodynamic forces.
2. The spontaneous generation of internal generalized thermodynamic flows resulting from these applied external generalized forces and the possibility of new internal forces that these flows themselves generate.
3. The potentiality of various distinct sets of these internal forces and flows for non-linear systems for the same initial and boundary conditions (i.e., multiple, locally stable, dissipative structures or processes, at stationary states)—each set of which can have a different rate of dissipation of the applied external potential (entropy production).
4. External or internal stochastic perturbations which, near a critical point, could cause the non-linear system to leave the local attractor basin in parameter space of one stationary state and evolve to that of another.
5. The non-deterministic (stochastic) tendency for evolution on perturbation to stationary states (dissipative structures) affording greater dissipation (entropy production), particularly through routes with autocatalytic and cross-catalytic steps, since these have a larger and thus more stable attractor basin in this generalized parameter space.

The following sections describe how the origin of life was a particular scenario of the carbon-based molecular dissipative structuring under the UV-C light potential of the early Archean, and how the continuation of this to the dissipative structuring of complexes of the fundamental molecules led to pigments absorbing visible wavelengths and their distribution over the entire surface of Earth, thereby increasing global photon dissipation.

## 4. Molecular Dissipative Structuring

The thermodynamics and dynamics of molecular dissipative structuring is similar to that of macroscopic dissipative structuring in that it involves the restructuring of material to foment the dissipation of an externally imposed generalized chemical potential. The difference is that it involves exciting intra-molecular degrees of freedom (rather than inter-molecular), producing structuring that foments the distribution of the conserved quantity (e.g., photon energy) over a greater number of microscopic degrees of freedom (e.g., a greater number of red-shifted photons). The macroscopic structures that arise are the concentration profiles of the different dissipatively structured molecules.

Soft UV-C photons arrived at the Earth’s surface throughout the Archean with an important integrated energy flux of ∼5 W m^−2^ [17,73]. Photons in this region of the spectrum have sufficient energy to re-configure covalent bonds of carbon-based molecules, but not enough energy to severely ionize and thereby dissociate or degrade them. The fossil record suggests that UV-C chromophores (now known as the fundamental molecules of life—Figure 1) were indeed the first pigments to appear.

The photochemistry of molecules in electronic excited states is much richer than the thermal chemistry of their ground state, because (1) the absorbed photon energy allows very endothermic reactions to occur, (2) anti-bonding orbitals can be reached, allowing reactions to occur which are prohibited in the ground state, (3) triplet states can be reached from the electronic excited state, allowing intermediates that cannot be accessed in thermal reactions, and (4) electronically excited molecules are often converted into radicals, making them much more reactive. For example, a molecule in its excited state can be a much stronger oxidizer or reducer with a substantially different pK_*a*_ value from that of its ground state (e.g., if the pK_*a*_ value becomes more acidic, proton transfer to an acceptor solvent water OH^−^ ion becomes much more probable). Singlet excited states have a particularly rich chemistry, while triplet states have a more restricted chemistry. This richness in photochemistry is, in itself, yet another strong argument in favor of the suggestion that the complex molecules of life arose out of photon-induced reactions occurring at the surface of the ocean rather than out of thermal reactions occurring at the bottom of the ocean.

The hallmarks of direct photon dissipative structuring [54] are:

1. Sufficient energy per photon to overcome activation barriers, as well as sufficiently large photoreaction quantum efficiencies.
2. A general increase in photon extinction coefficients and wavelength bandwidth as the molecules evolve from simple precursors towards final pigments [54].
3. The formation of conical intersections [15] connecting excited electronic states with the electronic ground state, allowing ultrafast (subpicosecond) radiationless dissipation (internal conversion).
4. A general trend towards dissipation of wavelengths of the prevailing surface solar spectrum of greater intensity.
5. Molecular ionization energies remaining greater than photon energies of the prevailing surface spectrum, thereby inhibiting photon-induced degradation.

Product molecules of dissipative structuring can (but not necessarily) have a lower Gibbs free energy than that of the precursor molecules from which they evolved. However, in thermal systems (chemical reactions), evolution to such a lower free energy state is not spontaneous if there are large energy barriers between configurations. Incident UV-C photons, on the other hand, allow coupling of the photon energy to the reactions (photochemical reactions), overcoming the barriers and even leading to higher Gibbs free energy configurations at a rate dependent on:

1. Photon intensities at the different wavelengths, I(λ).
2. The absorption cross-section of the molecule as a function of wavelength A(λ).
3. The widths of the phase-space paths leading to the particular conical intersection on the electronic excited state potential energy surface (i.e., the quantum efficiencies $q_{i}^{j}$) for particular molecular transformations i→j or internal conversion. Reverse transformations $q_{j}^{i}$, or transformations to other possible products (e.g., $q_{i}^{k}$), under UV light are less probable if the quantum efficiencies are smaller (smaller phase-space path on the excited potential energy surface) as compared to the quantum efficiency for internal conversion to the ground state $q_{j}^{IC}$.

**Figure 2:**
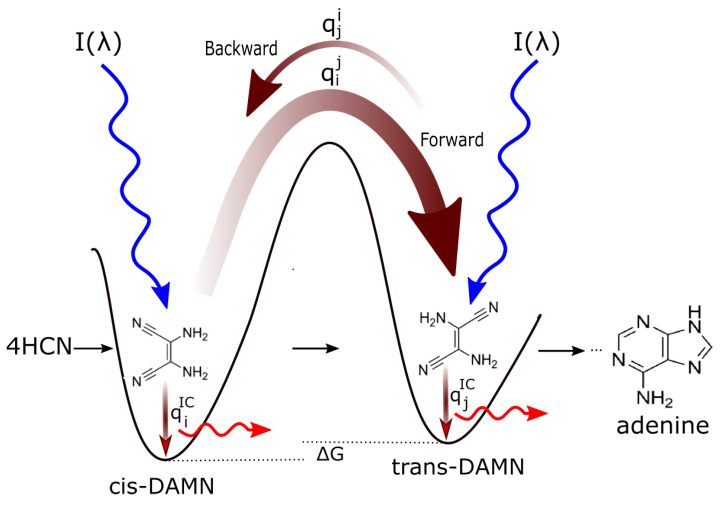
Mechanism for the evolution of molecular structures towards ever greater photon dissipative efficacy (molecular dissipative structuring) en route to the fundamental molecules (in this example adenine). The high activation barriers between configurations mean that reactions will not proceed spontaneously but only through coupling to photon absorption events. Forward and backward rates depend on photon intensities I(λ) at the different wavelengths of maximum absorption for the two structures and on the phase-space widths of paths on their excited potential energy surface $q_{i}$ leading to the conical intersection [15] giving rise to the particular transformation. This implies, in general, different quantum efficiencies for the forward ($q_{i}^{j}$) and backward ($q_{j}^{i}$) reactions as well as different quantum efficiencies for internal conversion ($q_{i}^{IC}$ and $q_{j}^{IC}$). Given different intensities of the incident spectrum at the absorption maxima, and since $q_{i}^{j}+\cdots q_{i}^{IC}=1$ and $q_{j}^{i}+\cdots q_{j}^{IC}=1$ (where “⋯” represents quantum efficiencies for other possible molecular transformations), those stationary states (concentration profiles) with greater photon dissipative efficacy (higher photon intensity at absorption maxima and higher quantum efficiency for internal conversion $q_{j}^{IC}$) will therefore gradually become dominant under a continuously impressed UV-C photon flux, independently of whether the change in the Gibbs free energy $\Delta G=G_{final}-G_{initial}$ of the molecule is negative or positive. This process of selecting molecular concentration profiles of ever greater photon dissipative efficacy, driving general evolution towards the right of the diagram, we call *natural thermodynamic selection*. Reproduced with permission from Michaelian [14].

Figure 2 explains this dissipative structuring (evolution) of the initial, poorly absorbing, precursor molecular concentration profile (HCN) towards concentration profiles of greater photon dissipative efficacy (including, finally, adenine) under the impressed soft UV-C photon spectrum of the Archean.

A similar process of selection on entropy production has been suggested by Hill in relation to inorganic morphological crystallization of a solid phase from a melt or solution. Dendritic or branched morphologies, which allow faster heat/mass dissipation, produce entropy at a higher rate than compact or planar morphologies and are the ones most likely observed [84]. Organic materials are, however, generally more effective at UV photon dissipation than inorganic materials since their vibrational modes in the electronic excited state can couple significantly to the vibrational modes of their electronic ground state through conical intersections (non-adiabatic coupling), unlike for most inorganic materials, except in special cases (e.g., near local defects in transition metal complexes [85]).

## 5. Examples of Molecular Dissipative Structuring

Hydrogen cyanide (HCN), cyanogen (NCCN) and carbon dioxide (CO_2_) are likely precursors of life’s fundamental molecules [86,87]. The Archean CO_2_ atmospheric concentration was likely 10 to 2500 times the modern concentration, with the lower limit being necessary to counteract the fainter young Sun for habitable surface temperatures [88]. HCN and NCCN are particularly important precursors for the nucleobases [14,35,57]. The formation of HCN and NCCN in the N_2_-rich atmosphere of the Archean required first breaking the triple covalent bond between nitrogens, N≡N. The $N_{2}$ photodissociation energy, ∼9.8 eV, corresponds to a wavelength of 126.5 nm (close to the solar Lyman-α line of 121.6 nm). Atomic nitrogen then attacks a carbon atom from CH or CH_2_ to form HCN [89]. Given the probably large Archean atmospheric abundance of N_2_ and CO_2_ and a carbon to oxygen ratio of C/O≥1, this is accomplished readily via photochemistry [90]. It has been estimated that HCN concentrations as high as $6\times 10^{-5}$ M might have been common in the enriched microlayer of the Archean ocean surface [14].

In previous articles [14,54,55,57] we have provided details of the UV-C photochemical molecular dissipative structuring of some of the fundamental molecules of life from common precursors in water. These articles can be consulted for a detailed analysis; here, only an outline is provided. There is a similarity in the dissipative structuring for three categories of the fundamental molecules (nucleobases, fatty acids, pigments), in spite of the diversity of their contemporary metabolic functions in life.

### 5.1. Nucleobases

**Figure 3:**
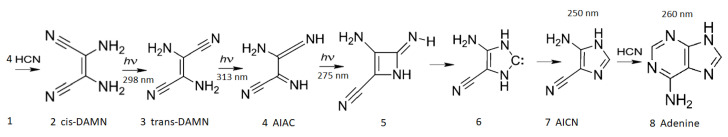
The photochemical synthesis of adenine from five molecules of hydrogen cyanide (HCN) in water, as discovered by Ferris and Orgel (1966) [35,51]. Four molecules of HCN are transformed into the smallest stable oligomer (tetramer) of HCN, known as cis-2,3-diaminomaleonitrile (cis-DAMN) (2), which, under a constant UV-C photon flux, isomerizes into trans-DAMN (3) (diaminofumaronitrile, DAFN). Further conversion on absorbing two more UV-C photons turns it into an imidazole intermediate, 4-amino-1H-imidazole-5-carbonitrile (AICN) (7). Hot ground state thermal reactions with another HCN molecule or its hydrolysis product formamide (or ammonium formate) lead to the purine adenine (8). This is a dissipative structuring process which ends in adenine, a pigment with a large molar extinction coefficient at 260 nm and a peaked conical intersection that promotes the dissipation of photons at the wavelength of maximum intensity of the Archean solar UV-C spectrum (Figure 1). Adapted from Ferris and Orgel (1966) [35].

The photochemical production of the nucleobase adenine from HCN in water has been studied experimentally by Ferrris and Orgel [35] and through time-dependent density functional theory by Boulanger [51]. In reference [14] we suggested that this was a molecular dissipative structuring process and identified the relevant photochemical reactions and demonstrated how photon dissipation increases with each step on route to adenine. We simulated the relevant photochemical and chemical reactions involved in the Archean dissipative structuring of adenine (Figure 3) occurring within a fatty acid vesicle floating on the ocean surface [14]. The process involves five molecules of hydrogen cyanide (HCN) in water which are converted in seven steps into adenine (Figure 3) under the soft UV-C spectrum (205–285 nm) of Figure 1.

**Figure 4:**
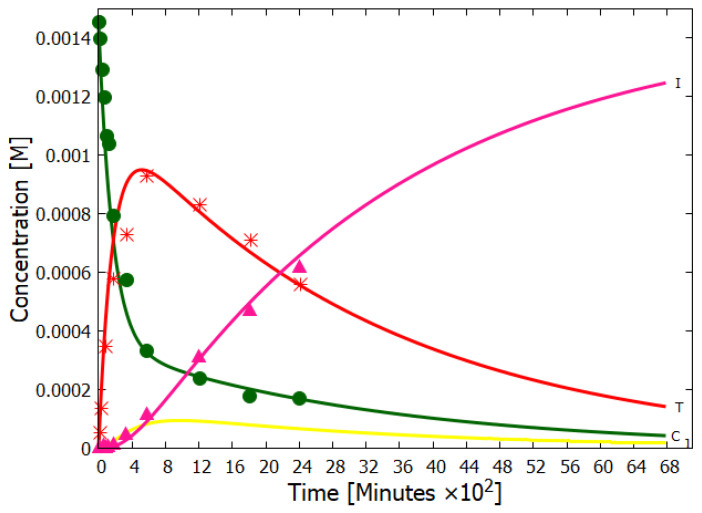
The concentrations of cis-DAMN (C, dark green), trans-DAMN (T, red), AIAC (J, yellow), and AICN (I, dark pink) obtained as a function of time from our simulation of the dissipative structuring of adenine compared with the experimental data points of Koch and Rodehorst (Figure 1 of reference [91]) starting with the same concentration of cis-DAMN as that in the experiment (0.00145 M). Two unmeasured quantum efficiencies and the unknown light intensity of the experiment (giving the time scale) were adjusted to give the best fit. The evolution of the concentration profile is such that the photon dissipation increases over time, a hallmark of molecular dissipative structuring (see Figure 18 of reference [14]), and in accordance with the non-equilibrium thermodynamic imperative. Reproduced with permission from Michaelian [14].

Details of our simulation of the dissipative structuring of adenine under UV-C light can be found in reference [14]. Here we only present our simulation results compared to the experimental data of Koch and Rodehorst [91] concerning the UV photo-transmutation of cis-DAMN (C) into trans-DAMN (T) and then into AIAC (J) and AICN (I) (Figure 1 of reference [91]), which are the important photochemical steps en route to adenine (Figure 3). This occurs through three photochemical reactions: $\gamma_{298}+C\to T$, $\gamma_{313}+T\to J$, $\gamma_{275}+J\to I$ (Figure 3). The results, plotted in Figure 4, show that our simulation, employing the initial experimental concentration of cis-DAMN (C) and the light conditions of experiment, reproduce very well the shapes of the three experimental data sets (see Michaelian [14] for details).

After each step en route to adenine from HCN, the global photon dissipation of the concentration profile of the different molecules involved increases (Figure 18 of reference [14])—a hallmark of dissipative structuring (Section 4). Adenine, the final product, has the largest photon absorption cross-section, peaking at 260 nm, exactly where the incident Archean spectrum (Figure 1) peaks, and a conical intersection for sub-picosecond dissipation of the electronic excitation energy into heat of the molecule and the local water environment.

### 5.2. Fatty Acids

In Section 5.1, the dissipative structuring of adenine was assumed to occur within a fatty acid vesicle floating at the Archean ocean surface. The existence of such vesicles, which spontaneously form from hydrocarbon chains (fatty acids) through Gibbs free energy minimization [92] or are photoinduced [93], is a common assumption in origin-of-life scenarios [94,95].

The mechanism usually proposed for fatty acid synthesis during the early Archean is that of heat-activated Fischer–Tropsch polymerization [96] of smaller hydrocarbon chains such as ethylene at the very high temperatures of deep ocean hydrothermal vents. A more likely scenario, assumed here, is that of the dissipative structuring of hydrocarbon chains under UV-C photons from CO_2_ or CO in water at moderate temperatures on the ocean surface [55]. Indications that ultraviolet light may have played an important role in the formation of hydrocarbons have come from a number of experiments. For example, it was shown in the early 1960s that irradiation of CO_2_ saturated water containing ferrous salts with UV-C light results in the production of formic acid and formaldehyde [97], while similar later experiments also produced methane [98] and ethane [99].

Fatty acid hydrocarbon tails can be extended through the sequential photon-induced polymerization of an initiator molecule such as ethylene, known as photo-polymerization. This occurs through direct photon-induced cleavage of the initiator molecule, controlled by a conical intersection [100], producing a free-radical which subsequently attacks the carbon–carbon double bonds of an existing polymer.

Saturated fatty acids do not absorb in the UV except for disassociation at <180 nm and a small peak at 207 nm due to the carboxyl head group absorption [101]. Under the Archean UV-C flux, photon-induced excited-state dehydrogenation or hydrogen bond proton transfer [102] could lead to a double carbon bond forming at any point on the hydrocarbon tail. A single double carbon bond in the tail will lead to absorption at ∼210 nm. Migration of the double bonds along the tail is known to occur [103], leading to conjugated bonds. The presence of two double bonds in a conjugated configuration (diene) gives strong absorption at ∼215–230 nm. Three conjugated double bonds (triene) will lead to absorption at ∼258–265 nm, while four double bonds (tetraene) will lead to absorption at ∼290–304 nm [104]. The diene and triene absorptions lie within the important UV-C spectrum arriving at Earth’s surface during the Archean (Figure 1).

**Figure 5:**
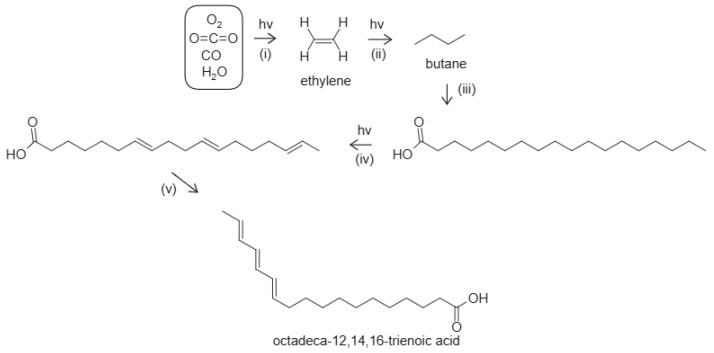
The photochemical dissipative structuring of an 18-carbon-atom fatty acid under UV-C + UV-B light. (i) UV-C-induced reduction of CO_2_ and CO in water to form ethylene, (ii) UV-C-induced polymerization of ethylene forming ever longer hydrocarbon chains, (iii) oxidation and hydrolysis stop the growing of the chain and form the carboxyl group, (iv) UV-C-induced deprotonation of the tail to form covalent double bonds, (v) double bond migration to a conjugated triene, octadeca-12,14,16-trienoic acid with a conical intersection. The final product absorbs strongly at ∼258 nm [104], near to the peak of the incident Archean UV-C spectrum at ∼260 nm (Figure 1).

The steps involved in the dissipative structuring of fatty acids are presented in Figure 5 and are summarized as follows:

1. UV-C-induced reduction of CO_2_ and CO in water saturated with these molecules to form ethylene.
2. UV-C-induced polymerization of ethylene to form long hydrocarbon tails with an even number of carbon atoms.
3. Oxidation and hydrolysis events to stop the growing of the chain and form the carboxyl group.
4. UV-C-induced excited-state dehydrogenation or hydrogen bond proton transfer of the tails to form a double bond.
5. Double bond migration to give a conjugated diene or triene with a conical intersection and strong absorption within the Archean UV-C spectrum.

Polymerization of ethylene occurs over a large UV wavelength region, but rates are more than two orders of magnitude larger at UV-C wavelengths (254 nm) than at UV-A (365 nm) [105]. Oxygen acts as a strong inhibitor to polymerization by rapidly reacting with the radical to form a peroxy-based radical which does not promote polymerization [105]. Such an oxidation reaction following hydrolysis is the origin of the carboxyl head group of the fatty acids. The presence of oxygen and the lack of surface UV-C light today mean that the hydrocarbon chain polymerization seen today at the ocean surface [106,107] is a poor remnant of what it probably was at the origin of life.

Hydrocarbons with conjugated dienes, trienes, or tetraenes almost always have conical intersections [108], allowing rapid dissipation of the electronic excited state energy. Reaching the conical intersection when in the electronic excited state involves twisting about two C=C bonds and decreasing one of the C-C-C angles, producing a temporary kink in the carbon backbone [108]. Therefore, as for the dissipative structuring of adenine, the same photons that dissipatively structured the fatty acid are the ones that will be dissipated efficiently by the final photochemical product (Figure 2).

These dissipatively structured conjugated fatty acids are, of course, robust to further photochemical reactions because of the sub-picosecond decay times of their electronic excited states (due to their conical intersections), which is too fast to allow for further appreciable photochemical transformation. They are thus the final molecular dissipative structures (one class of the fundamental molecules).

Within a wide range of pH values, fatty acids will form spherical vesicles through Gibbs free energy minimization. In order to maintain vesicle integrity at hot surface temperatures of ∼80–85 °C, prevalent (near the equator) during the early Archean, these fatty acids would have been long (∼18 carbon atoms) and cross-linked through UV-C light, which helps improve stability at high temperatures and over a wider range of pH values [55,109]. There is, in fact, a predominance of 16- and 18-carbon-atom fatty acids in the whole available Precambrian fossil record [110,111].

An additional advantage of vesicles, beyond serving as soft UV-C dissipators and promoting the internal concentration of larger synthesized molecules through semipermeability [14], is that they provide partial protection against occasional high-energy UV-C photons (<205 nm) through Mie scattering, thereby lowering the risk of ionization-induced molecular degradation [30].

### 5.3. Visible Pigments

The thermodynamic dissipation theory of the origin of life suggests that all fundamental molecules (common to all three domains) originated as molecular dissipative structures in the form of UV-C chromophores (pigments) [13]. These chromophores evolved individually and together to further increase photon dissipative efficacy and cover the entire solar spectrum. For example, the coupling of aromatic amino acids to their codons led to the nucleobases becoming information-carrying molecules, with this first information related to improving UV-C dissipative efficacy [61]. Another example is the fatty acids becoming vesicles, the protocell [55].

The thermodynamic imperative of increasing photon dissipation led to more complex biosynthetic pathways emerging for the dissipative structuring of pigments which absorb and dissipate photons of longer visible wavelengths of much higher photon intensity. Although these photons did not have sufficient energy individually for direct rearrangement of carbon covalent bonds, they provided sufficient energy for the phosphorylation of adenine to produce ATP [21,46,48,49,112], which could then, in numbers and in combination with cofactors and coenzymes, provide chemical potential for molecular transformation of covalent bonds.

We now consider the evolution through dissipative structuring of the most important pigment for life today in the visible—chlorophyll, the foundational molecule of contemporary visible photosynthesis [113]—and the contemporary production of ATP. The details of each step in the dissipative structuring of chlorophyll from the precursor L-glutamate on the ocean surface under UV-C light have been presented elsewhere [113]; here, only an overview is presented.

The pathway that produces chlorophyll a and b from glutamic acid in modern organisms [114] is proposed to have operated in the early Archean under UV-C photons, rather than relying on ATP and today’s complex enzymes. This route is Glutamic Acid → Glutamate-1-Semialdehyde (GSA) → 5-Aminolevulinic Acid (ALA) → Porphobilinogen (PBG) → Hydroxymethylbilane (HMB) → Uroporphyrinogen III → Coproporphyrinogen III → Protoporphyrinogen IX → Protoporphyrin IX → Mg-protoporphyrin → Mg-protoporphyrin monomethyl ester → Divinyl protochlorophyllide a → Monovinyl protochlorophyllide a → Chlorophyllide a → Chlorophyll a → Chlorophyll b.

**Figure 6:**
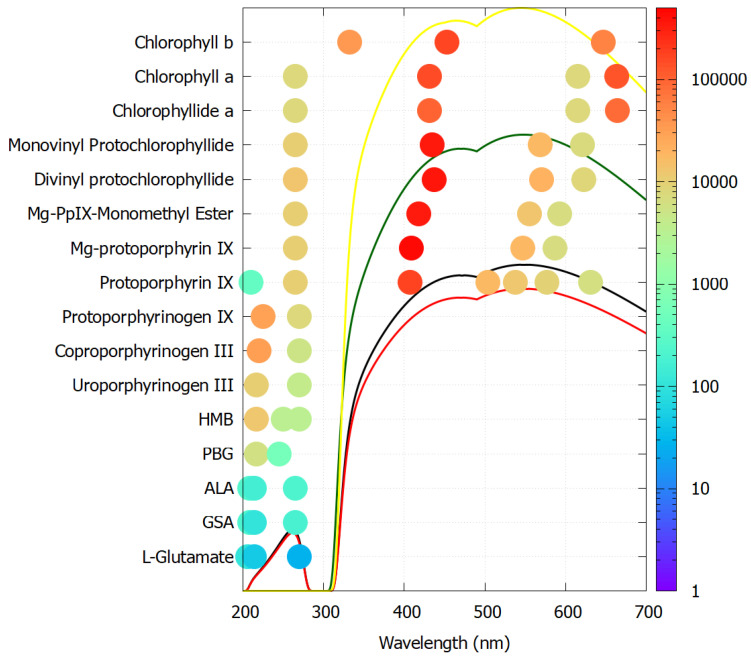
The molar extinction coefficients at peak wavelengths for all molecules en route to chlorophyll a and b. The color code on the right gives the size of the extinction coefficient in units of M^−1^ cm^−1^ and the location of the dots on the x-axis identifies the wavelengths of the resolved peaks. Porphobilinogen (PBG) and all later molecules have a conical intersection for extremely rapid internal conversion to the ground state. The black curve represents Earth’s surface solar spectrum at the origin of life (3.85 Ga), the red and green curves are the same for 2.9 Ga and 2.2 Ga, respectively, and the yellow curve is today’s surface spectrum. Reproduced with permission from Michaelian and Simeonov [113].

Figure 6 plots the molar extinction coefficients of all resolved peak absorption wavelengths for each molecule listed above en route to chlorophyll a and b. As predicted for molecular dissipative structuring, the extinction coefficients increase in size and the peaks increase in number and towards higher photon intensities as the molecule evolves from the precursor L-glutamate to the final chlorophyll molecule. Porphobilinogen (PBG) and all later molecules have a conical intersection for extremely rapid internal conversion to the ground state (photon dissipation into heat) [115].

At the intermediate protoporphyrin IX stage, absorption shifts into the visible range, while the 225 nm ultraviolet absorption band disappears. It is probable that at this point in evolutionary history, photosynthesis using visible light first became active, still based on photon dissipation but now through a more complex biosynthetic pathway using higher intensity visible light and prototypes of the known complex enzymes vulnerable to UV-C light (e.g., the D1 protein allowing water splitting) [116]. It was, therefore, probably around this time (∼2.4 Ga) that oxygenic photosynthesis began to saturate the oceans and atmosphere with oxygen, relegating UV-C dissipation to a protective ozone layer and thus allowing more complex biosynthetic pathways to arise on the surface, incorporating enzymes like D1 with weaker peptide bonding vulnerable in the 200–230 nm range.

## 6. The Fundamental Creative Force in Biology: Thermodynamic Selection of Dissipative Structuring

Darwinian natural selection cannot be viewed a fundamental creative force since it is not based on physical or chemical law. Natural selection is rather only a metaphor for thermodynamic selection, and useful only at the level of the organism. Application to other levels in the biological hierarchy leads to paradoxes and ambiguities at best, and complete failure at worst [19,53,117].

Perhaps one of the most conspicuous indications of the ineptness of Darwinian theory is that it sheds no light on the origin of life. Although efforts have been made to extend traditional evolutionary theory to include selection at the level of molecules, based either on their chemical or photochemical stability or their ability to sequester precursors (e.g., through chemical affinity), neither of these two lines of research have proven fruitful.

Non-equilibrium thermodynamic theory in the non-linear regime, on the other hand, as developed by Onsager, Machlup, Prigogine, Nicolis, Glansdorff, and many others ([2,6,83]), offers a physical and chemical description of the complex dynamics of a material interacting with its environment. Under this framework, processes (i.e., dissipative structures [2,3]) arise “spontaneously” under an external thermodynamic potential to dissipate this potential. Multiple, locally stable, *stationary states* exist for non-linear systems, and under perturbation, the system may evolve from one state to another, governed by both fluctuations (statistical) and thermodynamic law (deterministic).

The advantage of thermodynamic theory over traditional evolutionary theory is that it is based on established fundamental physical laws: the conservation laws, the second law of thermodynamics, and the continuity equations. Furthermore, the framework applies simultaneously to all biosystem levels, from fundamental organic molecules at the origin of life up to the present biosphere. Tautologies, paradoxes and stubborn problems inherent in Darwinian theory find resolution under this non-equilibrium thermodynamic framework, and a physical explanation for the origin, persistence, and evolution of life can be provided [19].

This section describes the evolution of carbon-based systems under thermodynamic selection of dissipative structures (or processes), based stochastically on increasing the global rate of dissipation of the prevailing solar spectrum (entropy production). Different mechanisms of thermodynamic selection are operative at the different biotic/abiotic levels, but they all are based on fomenting entropy production.

### 6.1. The Molecular Level

At the molecular level during the Archean (e.g., “The Pigment World” [52]), natural thermodynamic selection is that of molecular dissipative structuring as described above and presented in Figure 2. At this level and, in fact, at all levels, photon dissipative efficacy is selected and there is no correspondence with traditional Darwinian theory, which, in fact, fails here to provide a description of molecular selection, and, for that reason, offers no insights into the origin of life.

As an example, the dissipative structuring of adenine from HCN in water under UV-C light was outlined in Figure 2 and Figure 3 and described in detail in reference [14]. The final product, adenine, has a strong and wide photon absorption cross-section around the peak in the Archean surface spectrum at ∼260 nm (Figure 1) and a conical intersection for rapid internal conversion of the electronic excitation energy to heat, as do all the nucleobases.

As an example of increasing complexity at the molecular level, the aromatic amino acids tryptophan, tyrosine, phenylalanine and histidine absorb strongly close to the peak of the incident Archean UV-C spectrum (Figure 1) and do have conical intersections to internal conversion [29]. However, they also have significant quantum efficiency for fluorescence (e.g., tryptophan 0.13 and tyrosine 0.14 [118]), implying reduced efficacy for photon dissipation. Under TDTOL, and given these amino acids’ probable participation in a stereochemical era via their chemical affinity to DNA or RNA [119], the aromatic amino acids may have been UV-C antenna molecules for DNA and RNA, which have a much smaller quantum efficiency for fluorescence (e.g., adenine $2.6\times 10^{-4}$ [120]). Through fluorescent resonant energy transfer (FRET), the aromatic amino acids can pass their electronic excitation energy to the nucleic acids and thereby reduce their quantum efficiency for fluorescence by a factor of 10 [121,122], implying greater photon dissipation efficacy compared to the molecules acting separately. The decrease in quantum efficiency for fluorescence upon aromatic amino acid–nucleic acid interaction, in fact, is widely used to monitor protein binding to nucleic acid (e.g., via Stern–Volmer analysis [123]). The thermodynamic imperative of increasing entropy production thereby underlies the drive for increasing biotic complexity (e.g., amino acid–nucleic acid association) in order to increase photon dissipation, while also providing an explanation for the amino acid codon assignments [61].

Traditionally, aromatic amino acids have been considered as later additions to life’s repertoire due to their complex pathways in contemporary synthesis and because they are not found among the products of Miller-like experiments. However, this has been challenged by recent phylogenetic and ancestral sequence reconstruction studies indicating a pre-LUCA enrichment in aromatic amino acids [124]. Our TDTOL framework also suggests that the aromatic amino acids with their strong absorption around the 260 nm incident peak would be among the first to be dissipatively structured under UV-C light.

Another example of increasing complexity through increasing dissipation is the known stereochemical association of amphipathic (having both hydrophilic and hydrophobic portions) amino acids (tyrosine, tryptophan, methionine, lysine) with their codons [119] since these could serve as anchors, keeping the nucleic acid close to the ocean surface where UV-C light would have been most intense. Other characteristics of amino acids promoting photon dissipation through codon association are given in reference [61].

### 6.2. The Organism Level

At the level of the organism, thermodynamic selection resembles Darwinian natural selection; however, the criterion for selection is not the organism’s differential reproductive success given its environment, but rather the organism’s contribution to the global rate of entropy production of the ecosystem (or biosphere).

The mechanism of this can be elucidated with the following specific example: A wolf endowed with characteristics giving it success at the kill is therefore also successful at dissipating some of the chemical potential (free energy per unit mass) in the body of their prey, but more importantly, in spreading the non-utilized portion as excrement (fertilizer), serving as a catalyst for plant growth and thus greater global ecosystem photon dissipation. This was very apparent, for example, in the greening (and therefore greater photon dissipation) of Yellowstone National Park following the reintroduction of wolves in 1995 after their extinction by over-hunting in 1926 [125]. By increasing the conversion rate of prey to plant fertilizer, and by keeping the prey on the move (thus preventing overgrazing and spreading nutrients farther), successful wolves foment the global entropy production of their ecosystem. Wolves contributing less to entropy production will be those not as efficient at the kill. The mechanism by which these wolves are selected against is indeed their physical weakening through starvation, which correlates with a lower efficacy of reproduction, but selection fundamentally occurs due to the wolf’s contribution to the photon dissipation of its entire ecosystem.

At this level, the thermodynamic object of selection—differential contribution to global entropy production—closely correlates with Darwin’s proposed object of selection—differential reproductive success. However, the latter is only a poor proxy for the former. Selection in nature is based on a physical and, in principle, measurable global quantity—contribution to global biosphere entropy production (for example, photon dissipation in Yellowstone Park before and after reintroduction of the wolves), not on a local, tautological and in principle non-measurable quantity such as “differential reproductive success”. Long-term controlled experiments with bacteria indeed seem to demonstrate that global chemical potential dissipation, rather than local “fitness”, is optimized in nature [126].

### 6.3. The Ecosystem and Biosphere Levels

At the hierarchical level of ecosystems and the biosphere, parasitic, symbiotic, and mutualistic interactions among species from all three domains of life occur, as well as a coupling of biotic and abiotic dissipative processes. This foments solar photon dissipation from the ultraviolet well into the infrared through increasing the efficacy of plant and cyanobacterial growth, including the spread of organic pigments over the whole of Earth’s surface, thereby also catalyzing abiotic dissipative processes such as the water cycle, the carbon cycle, and ocean and wind currents [19,72,81,82]. Darwinian theory “explains” evolutive dynamics at this level as the emergent result of underlying interactions among all entities within the ecosystem. While not wrong, this explanation provides no insight into the direction of ecosystem evolution over time.

Others argue that Darwinian natural selection, in fact, does apply directly to higher-order entities, such as competition between species or clades. At these higher levels, however, competition loses significance since the number of entities in competition dwindles until reaching the limit of only one—the biosphere, which, itself, requires an explanation for its observed evolution [127].

Reference is also made to Gaia theory, where the Earth is considered as a self-regulating system in which interactions among entities, both biotic and abiotic, within the biosphere are selected such that this results in environmental conditions “favorable” to life in general [128]. However, here again, tautology arises through the introduction of the word “favorable”. More correctly, under the non-equilibrium thermodynamic perspective, a coupling of irreversible processes occurs through new dissipative structuring such that the global entropy production (solar photon dissipation) of Earth generally increases.

Similarly to thermodynamic selection at both the molecular and organism levels, selection at the biosphere level has both deterministic and stochastic elements. At the biosphere level, the existence of the system for a finite time in a particular thermodynamic stationary state—e.g., the climax state—out of many possible states in a non-linear system is contingent upon stationary state stability, and this, in turn, is contingent upon entropy production (as observed for abiotic thermodynamic stationary states). Internal or external fluctuations, macroscopic or microscopic, of any of the components of the biosphere which lead the biosphere to stationary states of greater global entropy production are the fluctuations most likely to be amplified (e.g., auto- or cross-catalytic or positive feedback processes).

In terms of thermodynamic forces and flows, a fluctuation in the biosphere may cause new thermodynamic forces to arise at any hierarchical level, giving rise to new generalized flows and the elimination of others. In this way, particular molecular concentration profiles, complexes of different molecules, individual organisms, communities, species, clades, ecosystems, and even biospheres arise, wax and wane, or go extinct accordingly.

**Figure 7:**
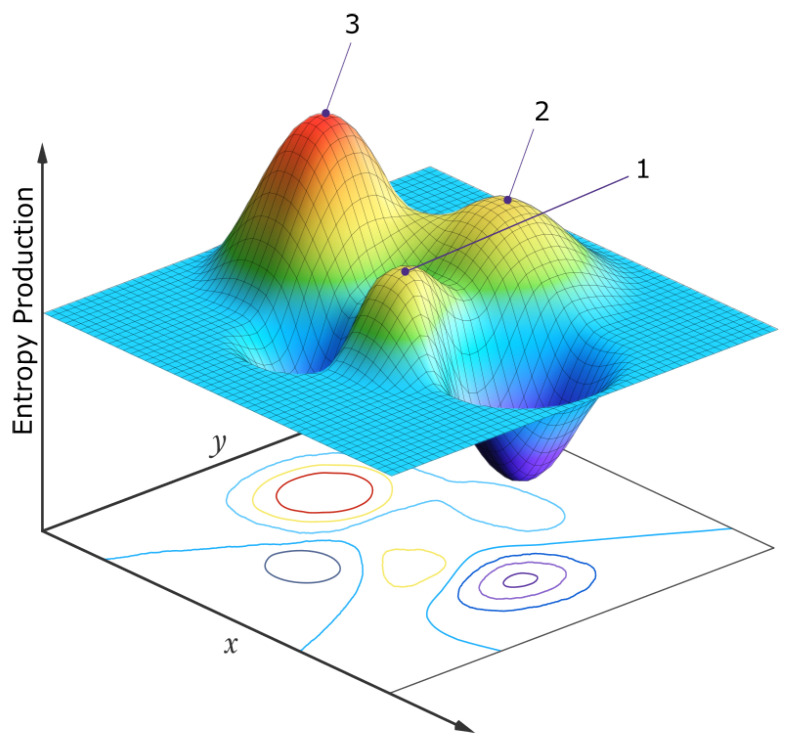
A simplified two-dimensional schematic representation of the entropy production surface (EPS) of a generalized phase space for a biosystem under a constant solar photon potential. The variables *x* and *y* at the origin of life may be, for example, the concentrations of different pigment molecules, while for an ecosystem today, the variables may be the populations of different species. Three locally stable stationary states at local peaks in the entropy production surface are presented. Following a large enough external or internal perturbation, the system evolves from one stationary state to another. Although fluctuations are generally stochastic, the system will most often be found in those stationary states with a larger attractor basin and generally with a higher peak in photon dissipation (the stationary state labeled “3”). For molecules, this corresponds to concentration profiles with greater quantum efficiency for dissipation to the ground state through a conical intersection. For an ecosystem, this corresponds to animal and plant population profiles giving greater total photon dissipation (*climax* ecosystems). If the system began in stationary state 1, its most probable future evolution would be 1 → 2 → 3, but any combination is possible. For the biosphere, the *x* and *y* variables might be the number of species in two different clades and sub-peaks corresponding to different species populations would exist on the main peaks, and evolution would usually be local, among the sub-peaks. However, every once in a while a perturbation may be large enough (for example, an asteroid impact) to move the system from one main peak to another (e.g., 1 → 3, mammals *y* becoming more prominent than dinosaurs *x*). Point, cyclic, or even chaotic dynamics are allowed to be superimposed on these peaks [78]. Autocatalytic stationary states have higher peaks and larger attractor basins in this generalized phase space and are thus more probable. The dimensionality of the generalized phase space is not fixed but evolves over time, providing new “shorter” routes to larger peaks of entropy production (e.g., the re-introduction of a population of wolves into the ecosystem of Yellow Stone National Park, see text). For a numerical analysis of a physical model depicting this, see reference [78]. Reproduced with permission from Michaelian [19].

Since selection is contingent upon global photon dissipation of the entire biosphere, at any particular hierarchical level, the biotic units do not compete with each other or struggle against their external environment, as imagined in the traditional Darwinian perspective, but rather form part of a quasi-stable global biotic–abiotic stationary state which “competes”, on stochastic fluctuation, with other similarly available stationary states of different photon dissipative efficacy in the neighborhood of a generalized phase space (e.g., molecular concentration space at the origin of life, or species population space at the level of today’s ecosystems). Stationary states, under the specific environmental conditions (the solar photon potential), that result in greater photon dissipation are generally more probable since they have a larger/stable attractor basin and greater photon dissipation in this space (Figure 7).

Finally, since today’s biosphere has both biotic and abiotic components coupled on many different hierarchical levels and over different time scales, it is relevant to make a few remarks concerning the coupling of biological irreversible processes with abiotic dissipative processes and the plasticity (adaptability) of this coupling over time. For example, the water cycle is coupled to the heat of dissipation of photons in organic pigments in the leaves of plants or within cyanobacteria on the surfaces of the oceans, lakes, and wet soils. The coupling of the water cycle to photon dissipation in organic pigments is autocatalytic, since more water in the water cycle means a greater greening of Earth, which implies more water in the water cycle [82,129]. The water cycle dissipates the infrared light resulting from the heat of photon dissipation in the leaves and cyanobacteria even further towards the infrared, finally emitting the energy into space at the cloud tops having an approximate black-body temperature of −14 °C (giving an emission peak at ∼11 μm).

Both biotic organisms and abiotic processes have the ability to adapt to a changing impressed thermodynamic potential. Biotic organisms today adapt through their organismal plasticity (e.g., the ability to migrate, the ability to survive off different thermodynamic potentials—heterotrophy—or through mutation of their genes and reproduction—plasticity at the species level). In contrast, abiotic processes have an inherent plasticity, for example, a change in size or direction of a hurricane in response to a change in the ocean surface temperature. A hurricane is, in fact, steered by ocean surface cyanobacterial pigment concentration dissipating the sunlight into heat [130], yet another biotic–abiotic coupling increasing dissipation.

## 7. Conclusions

Dissipative structuring in biology has been ongoing, from its first appearance as molecular dissipative structuring of the fundamental molecules (pigments) under the UV-C surface light of the Archean to today’s coupling of biology with abiotic dissipative structures. Strong broadband absorption of the fundamental molecules of life in the UV-C was not required for photoprotection, nor for primordial synthesis, but it was for photodissipation. Today, the sum of these structures or processes makes up the global dissipative process known as the biosphere, which dissipates the entire solar spectrum well into the infrared. Earth emits the incident high-energy solar photons as many more low-energy photons in the form of far-infrared light with wavelengths between ∼8 and 14 μm, giving Earth an entropy production per unit area and per photon of almost twice that of its lifeless neighboring planets.

Neo-Darwinian evolutionary theory proposes that the creative force in biology is natural selection through the organism’s struggle against an imposing external environment and competing organisms. Gaia theory speaks of “life (collectively) shaping the environment for its mutual benefit”. Both descriptions are tautological and ambiguous. The non-equilibrium thermodynamic perspective, on the other hand, suggests that irreversible processes such as life are instead dissipative structures or processes arising “spontaneously” to dissipate impressed generalized thermodynamic potentials (principally the solar photon potential). These processes are thermodynamic flows which rise and fall in response to changes in internal thermodynamic forces. Fluctuations near critical points take the global system to different stationary states (sets of quasi-stable forces and flows) and in general toward greater stability, corresponding to increasing solar photon dissipation, as depicted in Figure 2 for the fundamental molecules at life’s origin and in Figure 7 for today’s biosphere.

The fundamental creative force in biology is thus thermodynamic and gives rise to two categories of structures: equilibrium and non-equilibrium structures. The spontaneous formation of these categories of structures involves the variables of entropy and entropy production, respectively. The general trend for equilibrium systems is towards maximum entropy, while for non-equilibrium systems in the non-linear regime, it is towards greater global entropy production. The evolution over time of structures or processes, in both cases, can be described by physical/chemical principles derived from the conservation laws, the second law of thermodynamics, and the continuity relations.

## Data Availability

No new data were created.

## Abbreviations

The following abbreviations are used in this manuscript:

ALA	5-Aminolevulinic Acid
ATP	Adenosine triphosphate
CIT	Classical Irreversible Thermodynamic theory
CO_2_	Carbon dioxide
DNA	Deoxyribonucleic acid
GSA	Glutamate-1-Semialdehyde
H_2_S	Hydrogen sulfide
HCN	Hydrogen cyanide
HMB	Hydroxymethylbilane
LOV	Light–Oxygen–Voltage—organisms’ blue-light-sensing protein modules
PBG	Porphobilinogen
RNA	Ribonucleic acid
SO_2_	Sulfur dioxide
TDTOL	Thermodynamic Dissipation Theory of the Origin of Life
UV-A	Light within the 315–400 nm region
UV-B	Light within the 280–315 nm region
UV-C	Light within the 100–280 nm region
UV-C (hard)	Light in the 100–205 nm region
UV-C (soft)	Light within the 205–285 nm region
LUCA	Last universal common ancestor
